# Development of a Flexible Metamaterial Film with High EM Wave Absorptivity by Numerical and Experimental Methods

**DOI:** 10.3390/ma15124133

**Published:** 2022-06-10

**Authors:** Chin-Hsiang Cheng, Yi-Shen Chen, Hsin-Yu Tsai, Yu-Ling Liang, David T. W. Lin, Yitung Chen

**Affiliations:** 1 Department of Aeronautics and Astronautics, National Cheng Kung University, Tainan City 70101, Taiwan; p48981081@mail.ncku.edu.tw (Y.-S.C.); jocelyn380@yahoo.com.tw (H.-Y.T.); p46104120@gs.ncku.edu.tw (Y.-L.L.); 2 Institute of Mechatronic System Engineering, National University of Tainan, Tainan City 700301, Taiwan; david@mail.nutn.edu.tw; 3 Department of Mechanical Engineering, University of Nevada-Las Vegas, Las Vegas, NV 89154, USA; yitung.chen@unlv.edu

**Keywords:** flexible metamaterial structure, manufacturing, numerical simulation, experiments, electromagnetic wave absorption

## Abstract

The present study is intended to develop and test a cost-effective and efficient printing method for fabricating flexible metamaterial film with high electromagnetic wave absorptivity. The film can be easily applied to the surfaces with curved aspects. Firstly, numerical parametric study of the absorption characteristics of the film is performed for the range of frequency varying from 2.0 to 9.0 GHz based on commercial software package. Secondly, the flexible metamaterial films are fabricated, and experiments are conducted. The flexible metamaterial film consists of a flexible dielectric film made of polyimide (PI) and an array of split-ring resonators. The split-ring resonators of different geometric dimensions are fabricated on the PI film surface by using a silver nanoparticles ink jet printer. The performance of the flexible structure is then measured and dependence of operation frequency with higher absorptivity on the dimensions of the split-ring resonators is investigated. A comparison between the numerical and experimental data shows that the numerical predictions of the operation frequency with higher absorptivity closely agree with the experimental data.

## 1. Introduction

Metamaterials are unique structures which have negative permittivity and negative magnetic permeability, as originally described by Veselago [1]. Their electromagnetic wave absorption may reach a high value if the structures are properly designed. A metamaterial absorber mainly consists of three layers: (1) an array of periodically arranged metallic patterns, (2) a dielectric layer and (3) a continuous metallic layer. The array of metallic patterns is used for minimizing the reflectance of electromagnetic (EM) waves by impedance matching with incident medium. By placing the array of metallic patterns on the dielectric layer, both negative permittivity and negative permeability may be yielded. The continuous metallic layer is used to hinder the transmission. However, if the transmission is allowed, the continuous metallic layer may not be necessary. The characteristic length of the structure has a subtle influence on the wavelength of the absorbed electromagnetic wave. The complex structure may then be treated as a homogeneous medium based on the effective-medium theory [2], with which the effective EM properties can be determined. Numerous existing studies, to name a few, Shelby et al. [3] and Cho et al. [4], have already proven that the native refractive index can indeed be realized practically by implementing a periodic metallic array on semiconductor.

Metamaterials can be applied in a number of engineering applications such as EM wave absorber [5,6,7,8], electromagnetic wave cloaking [9], super-lenses [10], filter [11], antenna [12], shielding the electromagnetic wave [13], solar cell [14], and so on. The feature of increasing the electromagnetic wave absorption can be employed in stealth technology, and the shielding effects is critical to the protection of human body from the electromagnetic field in natural environment. Winson et al. [5] studied a hybrid broadband radar absorber by using metamaterial and graphene. Authors performed numerical simulation of the EM wave from 1 to 60 GHz, and they also conducted experiments on the absorptivity from 9.9 to 10.2 GHz and found an absorptivity of 96.05%. Mondal et al. [13] designed a structure of metamaterial which can used in microwave shielding applications. Their simulation results showed strong electromagnetic interference shielding effectiveness (SE). One of their test cases reached 99% in absorptivity of the polluting electromagnetic wave in the X- and Ku-band frequency ranges.

On the other hand, it has been recognized that the geometry of the periodic metallic array patterns exhibits remarkable effects on the operation frequency and the absorptivity. Therefore, the attention of the related researchers has been drawn to the effects of a variety of the geometry such as split-ring resonators [15,16], flower-shaped structure [17], fishnet-shaped structure [18,19], lumped elements [20] and many more [21,22,23].

It is also important to note that in these existing reports [3,4,5,6,7,8,9,10,11,12,13,14,15,16,17,18,19,20,21,22], the metamaterials structures were basically nonflexible such that they could not be applied to the surfaces with curved aspects. Unfortunately, only a few studies took the flexible structure into account. For example, Yoo et al. [23] used a planar and flexible dielectric Teflon layer to make the metamaterial and obtained the low-frequency perfect absorption with very small unit-cell size in snake-shape structure. Zheng et al. [24] developed a flexible metamaterial absorber with two resonators and four resonances located in GHz and THz ranges. They used a sandwich microstructure consisting of periodic metallic patches on a metasurface, a dielectric of FR4 board on the interlayer, and a continuous copper film on the substrate. More recently, Zhao et al. [25] developed a transparent flexible metamaterial absorber (MMA), consisting of a multilayered structure with a transparent polyvinyl chloride layer and a periodic indium-tin-oxide patch array attached to a polyethylene terephthalate film layer.

A metamaterial absorber is a type of metamaterial intended to efficiently absorb electromagnetic radiation, as has already been discussed in Refs. [1,2,3,4,5,6,7,8,9,10,11,12,13,14,15,16,17,18,19,20,21,22,23,24,25]. The metamaterials are those in which the resonant state with significant changes in electromagnetic responses and thus producing extreme values of effective permittivity and permeability within a range about the resonant frequency. Such metamaterials enabled advancements in the fields of electromagnetics. It has been found that the effective permittivity and permeability can be engineered to create high absorption by manipulating resonances to absorb both the incident electric and magnetic field. The present study aims to develop a cost-effective and efficient fabrication method for the flexible metamaterial structures with high electromagnetic wave absorptivity. The structure mainly consists of a flexible dielectric layer made of polyimide (PI) and an array of split-ring resonators. The split-ring resonators of different geometric dimensions can be easily printed on the PI layer by using a silver nanoparticles ink jet printer. Therefore, the costly fabrication process for the split-ring resonators can be replaced with the printing method. Numerical simulation and experiments on the absorption characteristics of the film are performed within the range from 2.0 to 9.0 GHz.

## 2. Flexible Metamaterial Film with Array of Silver Split-Ring Resonators

**Figure 1:**
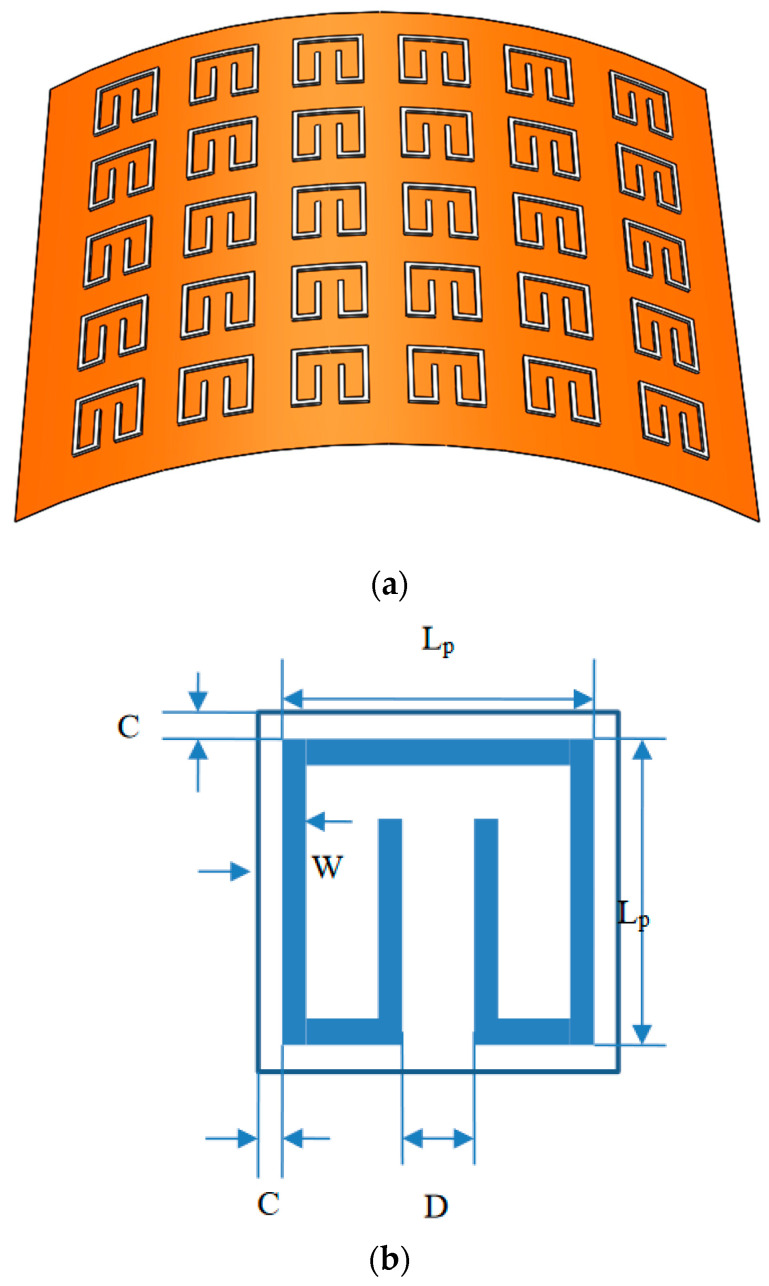
Flexible metamaterial structure. (**a**) Metamaterial film, (**b**) Unit cell and its dimensions.

The flexible metamaterial film with array of silver split-ring resonators is shown in Figure 1a. The silver rectangle-shaped patterns are printed on one surface of the PI layer. The thickness of the PI film is 125 m, and the thickness of the silver patterns 17 μm. Figure 1b illustrates a unit cell and the notations of its dimensions. The array is arranged with a spacing of 2C between two patterns and the size of each pattern is L_p_ × L_p_. The thickness of the wire is W and the split gap D. The geometric parameters (L_p_, C, D and W) of the structure are adjusted in a numerical parametric study to achieve higher absorptivity.

## 3. Numerical Methods

The Materials and Methods should be described with sufficient details to allow others to replicate and build on the published results. Please note that the publication of your manuscript implies that you must make all materials, data, computer code, and protocols associated with the publication available to readers. Please disclose at the submission stage any restrictions on the availability of materials or information. New methods and protocols should be described in detail while well-established methods can be briefly described and appropriately cited.

### 3.1. Mathematical Equations

The numerical simulation is performed based on a commercial software package. However, in this section the mathematical equations are described briefly. The relation between permittivity and permeability are described with dispersion relation [26]. In electromagnetic theory, these two basic parameters are essential to the propagation of the electromagnetic waves, which can be governed by the Maxwell’s equations which combining the Faraday’s law, Ampere’s law, Gauss’s law and Gauss’s law for magnetism as

(1) $$\nabla \times \vec{E}=-\frac{\partial \vec{B}}{\partial t}$$

(2) $$\nabla \times \vec{H}=\vec{J}+\frac{\partial \vec{D}}{\partial t}$$

(3) $$\nabla \cdot \vec{D}=\rho$$

(4) $$\nabla \cdot \vec{B}=0$$

where $\vec{E}$ is the electric field intensity (V/m), $\vec{D}$ the electric flux density (C/m^2^), $\vec{H}$ the magnetic field intensity (A/m), $\vec{B}$ the magnetic flux density (T), $\vec{J}$ the current density (A/m^2^) and ρ the electric charge density (C/m^3^).

To understand electromagnetic phenomena, it is essential to solve Maxwell’s equations numerically. In the frequency domain, Maxwell’s equations reduce to

(5) $$\nabla \times \mu_{r}{}^{-1}\left(\nabla \times \vec{E}\right)-k^{2}\left(\varepsilon_{r}-\frac{i\sigma }{\omega \varepsilon_{0}}\right)\vec{E}=0$$

where ω is frequency of wave, k wave number determined with $k=\omega \sqrt{\varepsilon_{0}\mu_{0\;}}$ =  ω/c, in which $\varepsilon_{0}\;$ is the vacuum permittivity, $\mu_{0}$ is the vacuum permeability and *c* is the speed of light which equals 2.9979 × 10^8^ m/s. In addition, $\varepsilon_{r},\;\;\mu_{r},$ and σ are the relative permittivity, relative permeability, and electrical conductivity, respectively. For a simple two-dimensional problem, theoretical approach for analytically solving the above equations was presented in two dimensions by Gric et al. [27]. However, in the present three-dimensional simulation, a three-dimensional finite element frequency domain analysis for solution of Equation (5) is performed based on the framework of a commercial software package, COMSOL.

### 3.2. Physical Model and Boundary Conditions

**Figure 2:**
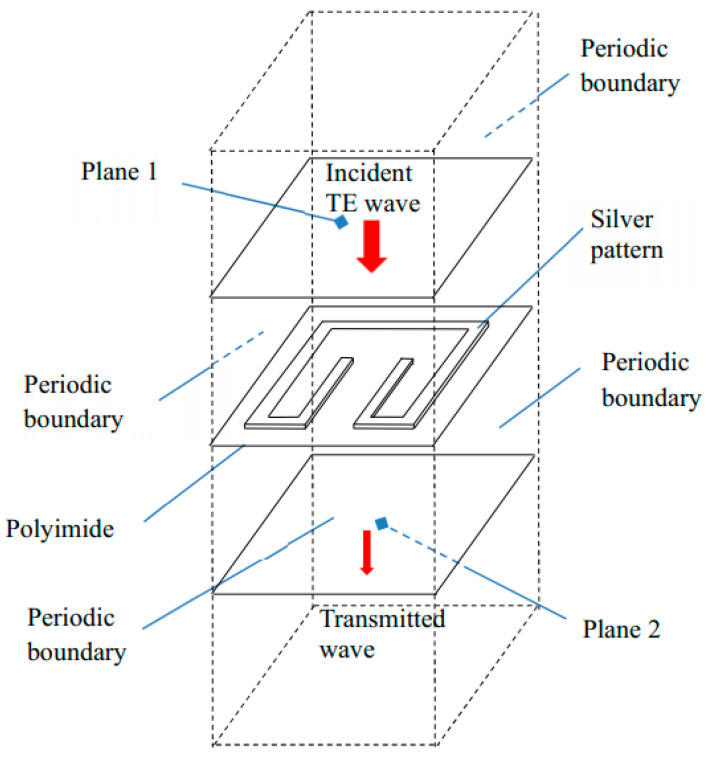
Solution domain.

Figure 2 shows the physical model and the boundary conditions. To simplify the model, the solution domain includes only a unit cell of metamaterials and the upper and lower air spaces. As plotted in in Figure 2, Plane 1 is set to be the top incident surface where an incoming 1-W TE wave is emitted toward the cell. Plane 2 is located beneath the unit cell which receives the transmitted electromagnetic energy.

The vertical faces around the structure are set to be Floquet periodic boundaries such that the waves are transposed from the source to destination boundary with the appropriate phase shift. The unit cell of metamaterial is placed on the polyimide film. The direction of the incident waves is normal to the metamaterial surface. The electric and magnetic field can be discretized into two polarizations with field normal to the boundary. Continuity boundary condition is applied at all inner interfaces.

S-parameters are calculated and used as measure of reflected power and transmitted power in a network as a function of frequency.

(6) $${\bar{S}}_{11}=\frac{\sqrt{E_{1,r}}}{\sqrt{E_{1,i}}}$$

(7) $${\bar{S}}_{21}=\frac{\sqrt{E_{2,t}}}{\sqrt{E_{1,i}}}$$

where ${\bar{S}}_{11}$ is the reflection coefficient and ${\bar{S}}_{21}$ is the transmission coefficient, in which $E_{1,i}$ is power emitted from plane 1, $E_{1,r}$ is power reflected from metamaterial structure and back to plane 1, $\mathrm{and}\;E_{2,t}$ is power transmitted through metamaterial and received by plane 2. By Kirchhoff’s radiation law, absorptivity (α) in this case is determined by

(8) $$\alpha \left(\omega \right)=1-{\left|{\bar{S}}_{11}\right|}^{2}-{\left|{\bar{S}}_{21}\right|}^{2}$$

For a metamaterial having a third continuous metallic layer that will block the EM wave and result in no transmission through the structure, $\left|{\bar{S}}_{21}\right|\to 0$, and hence

(9) $$\alpha \left(\omega \right)=1-{\left|{\bar{S}}_{11}\right|}^{2}$$

### 3.3. Numerical Parametric Study

**Figure 3:**
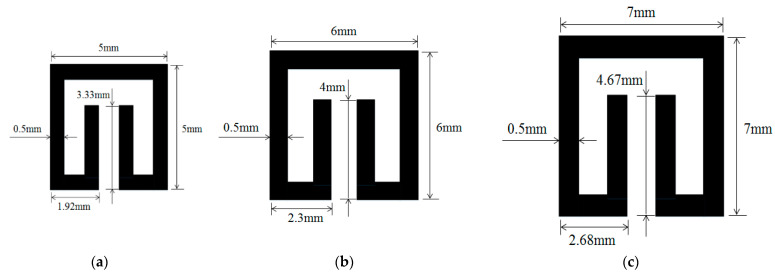
Dimensions of three tested split-ring resonators. (**a**) SRR1, (**b**) SRR2, (**c**) SRR3.

The effects of geometric parameters (L_p_, C, D and W) are evaluated by using the numerical method. Dependence of absorptivity on frequency at specific combinations of the geometric parameters is investigated. Herein, three baseline split-ring resonators (SRR1, SRR2 and SRR3) are considered firstly, and their dimensions are given in Figure 3. The sizes of the test cases are assigned to be 5 mm with SRR1, 6 mm with SRR2 and 7 mm with SRR3.

**Figure 4:**
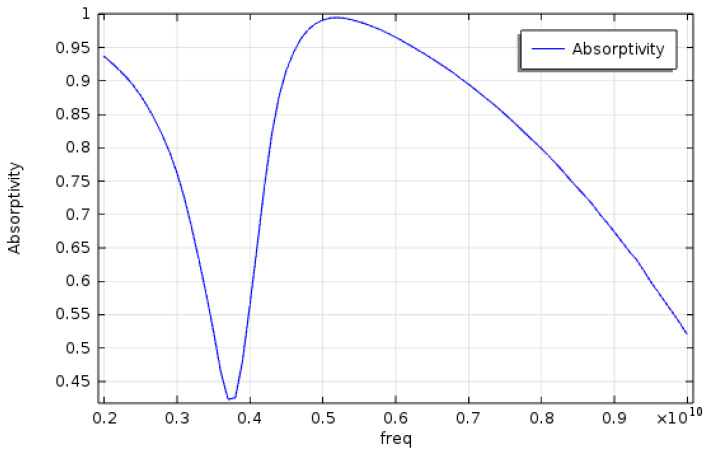
Absorptivity vs frequency, for SRR2.

Figure 4 shows the dependence of absorptivity on the frequency of the EM wave for case SRR2. In the present study, Equation (8) is used to calculate the absorptivity of the metamaterial structure. It is found that for this case the maximum absorptivity reaches 0.99 at around 5.3 GHz and minimum absorptivity 0.43 at 3.7 GHz. Apparently, for SRR2, the operation frequency ought to be 5.3 GHz. Furthermore, similar analysis depicts that the operation frequencies for SRR1 and SRR2 are 6.5 GHz and 4.3 GHz, respectively.

To obtain a deeper insight into the electromagnetic fields for the three baseline cases at their respective operation frequencies, Table 1 conveys the distributions of the magnitudes of electric field intensity, magnetic field intensity, and magnetic energy density for SRR1 at 6.5 GHz, SRR2 at 5.3 GHz, and SRR3 at 4.3 GHz individually. This table basically provides the numerical predictions of effects of size (L_p_) on the electromagnetic resonance in the metamaterial structures. It is noted that for all the cases, higher electric field intensity is found within the slit at the center. It is because the effect of electric field concentration occurs within the slit, mainly in the dielectric layer, and the free electrons in the conductor collectively resonate and couple with the external electromagnetic wave to generate an enhanced electric field. As the size of the split-ring resonator becomes smaller, both the intensities in electric and magnetic fields become higher. Furthermore, stronger magnetic field intensity or energy is distributed along the perimeter of the rectangle. The distribution of magnitude of magnetic field intensity is bilaterally symmetric with respect to the vertical central line and nearly forms a cycle in magnetic field.

Next, the parametric study is extended to cover more general cases. Table 2 displays a part of numerical predictions of the operation frequency with different combinations of the geometric parameters. Note that in the parametric study, as one parameter is varied, the other three parameters are fixed. The effects of each geometric parameter can be observed according to the numerical data provided. It is found that an increase in L_p_ leads to a decrease in the operation frequency. However, on the contrary, the operation frequency increases when the dimension of C, D or W is elevated. For the cases considered in this table, the operation frequency ranges between 4.2 and 5.8 GHz. It is noticed that an adjustment in the geometric parameters could alter the operation frequency of the metamaterial structure. The dependence of operation frequency with higher absorptivity on the dimensions of the split-ring resonators is actually essential for optimization of the patterns. However, since the combined influence of the geometric parameters is rather complicated, further study of this issue is still necessary.

## 4. Experimental Methods

**Figure 5:**
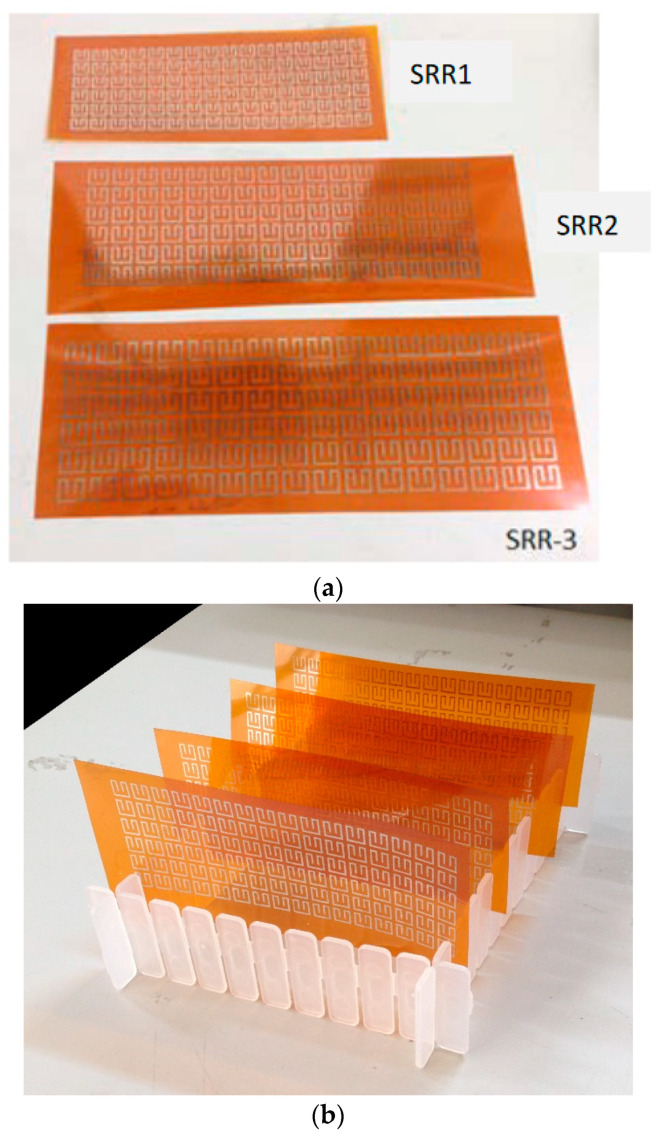
Photographs of printed split-ring resonators. (**a**) SRR1, SRR2, and SRR3, (**b**) More printed products.

The split-ring resonators of different geometric dimensions are fabricated on the PI film surface by using a silver nanoparticles ink jet printer. When necessary, an electromagnetically transparent protective layer may be sprayed on the surface for resisting oxidation, abrasion and dirt. The thicknesses of the PI film and the split-ring resonators are 125 and 17 μm, respectively. The temperature-resistant PI film is able to endure a temperature up to 400 °C. Figure 5 gives the photographs of the fabricated products. The three baseline split-ring resonators are shown in Figure 5a.

**Figure 6:**
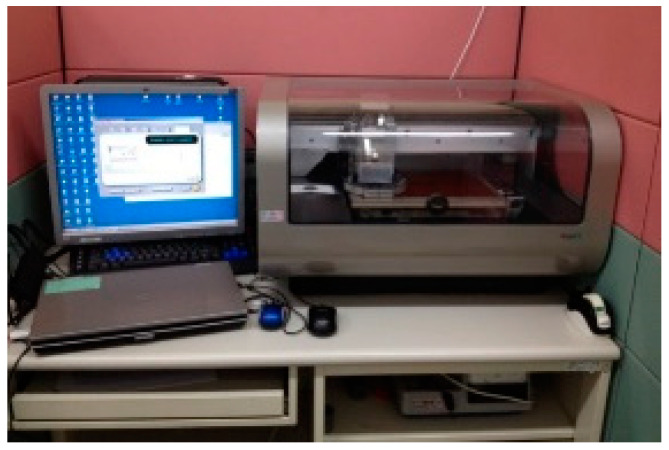
Silver nanoparticles ink jet printer.

The silver nanoparticles ink jet printing process is selected in this study since it is efficient, simple, and inexpensive for printing the patterns on flexible materials such as PI film. Besides, during the printing process no chemical waste is generated, and thus no post-processing costs are required. The silver particle agglomeration can be improved by using dispersants, surfactants, and polymers in order to have a jetting of high quality. The patterns are fabricated by inkjet printing technique using Fujifilm Dimatix printer (DMP 2850) shown in Figure 6. The printer uses piezoelectric inkjet technology which allows depositing fluids materials on substrate. The silver nanoparticle ink is required to be sonicated around 15 min to avoid the agglomerate between the particles. The fluid (~3 mL) is then placed into the cartridge with a syringe, a needle and 0.2 m filter. The filter is used to filter out the particles that are bigger than 0.2 m to prevent the nozzle head clog.

**Figure 7:**
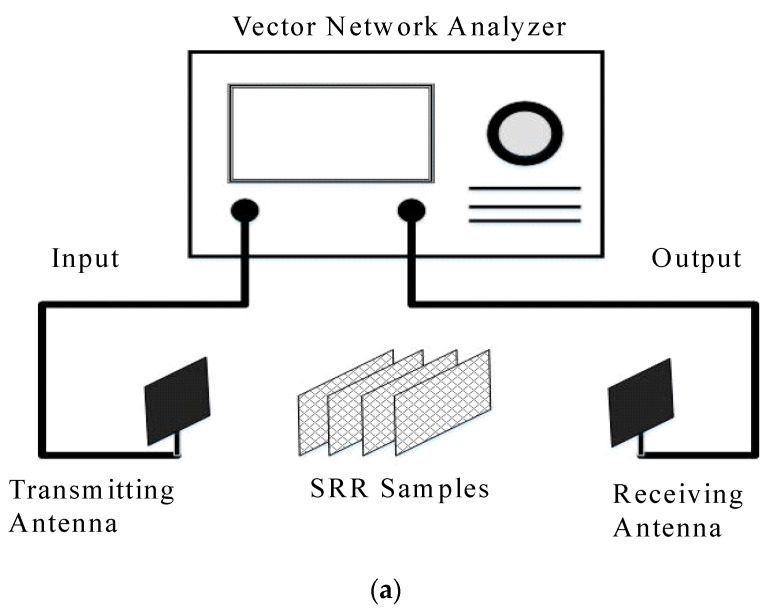

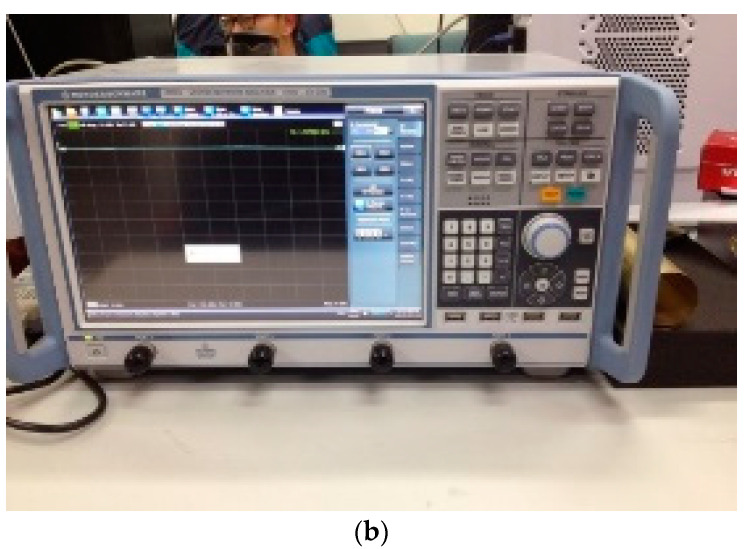
Experimental apparatus. (**a**) Schematic of experimental system, (**b**) Vector network analyzer.

Schematic of the experimental system is illustrated in Figure 7a and the photograph of the vector network analysis is given in Figure 7b. The vector network analyzer, Rohde & Schwarz ZNB8 VNA, which produces microwave in the frequency range of 2~8.5 GHz is adopted to measure the network scattering parameters (S-parameters). The equipment enables the RF transmission between the transmitting and the receiving antennas. The SRR specimens are placed in between the two antennas. Note that getting the calibration plane dialed in correctly is the most crucial step in matching the network design. A poor calibration can result in significantly different results. The characteristics of the three fabricated SRRs are then measured and dependence of operation frequency with higher absorptivity on the dimensions of the split-ring resonators is investigated. Due to the limitation of the experimental system, the experimental chamber is not isolated perfectly and hence, over the air (OTA) testing is conducted to find the operation frequency with maximum absorptivity.

The measured quantity from the vector network analyzer is the transmission coefficient, ${\bar{S}}_{21}$. Then, based on the obtained results of ${\bar{S}}_{21}$, a logarithmic transmission coefficient, $S_{21}\;$ can be further obtained as

(10) $$S_{21}=20\log|{\bar{S}}_{21}|$$

where $S_{21}\;$ is in decibels (dB).

## 5. Experimental Results and Discussion

**Figure 8:**
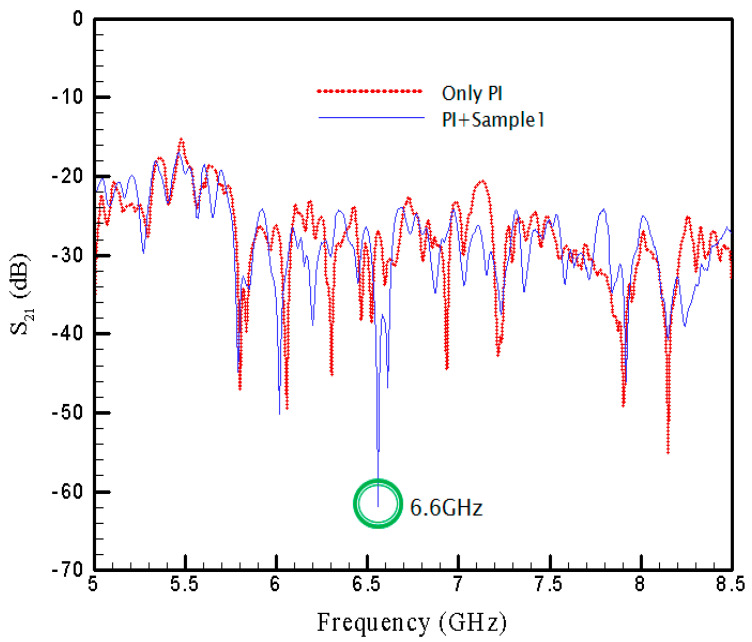
Transmission coefficient with SRR1.

Figure 8 shows the experimental results of *S*_21_ versus frequency for the case SRR1, whose size is 5 mm, under the inevitable indoor noise. Nonetheless, in this figure the transmission coefficient for the PI film without the pattern array is also provided for reference. In comparison between the two curves with and without the printed patterns, one can find a significant drop for the curve with SRR1 at 6.6 GHz. As the value of *S*_21_ at this frequency is introduced into Equations (8) and (10), one can obtained the maximum absorptivity of 97.45%.

**Figure 9:**
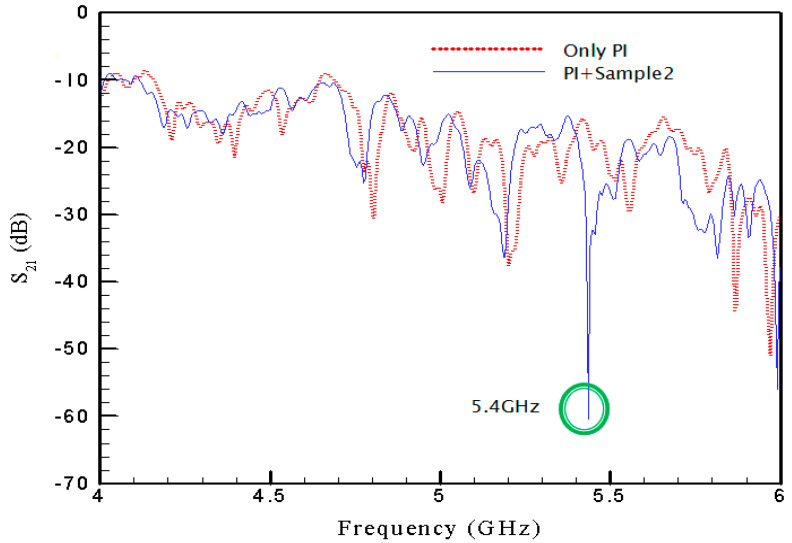
Transmission coefficient with SRR2.

Plotted in Figure 9 is the transmission coefficient for SRR2 with pattern size of 6 mm. For this particular case, the negative peak of *S*_21_ is found at frequency of 5.4 GHz. The negative peak transmission coefficient yields a maximum absorptivity of 96.6%.

**Figure 10:**
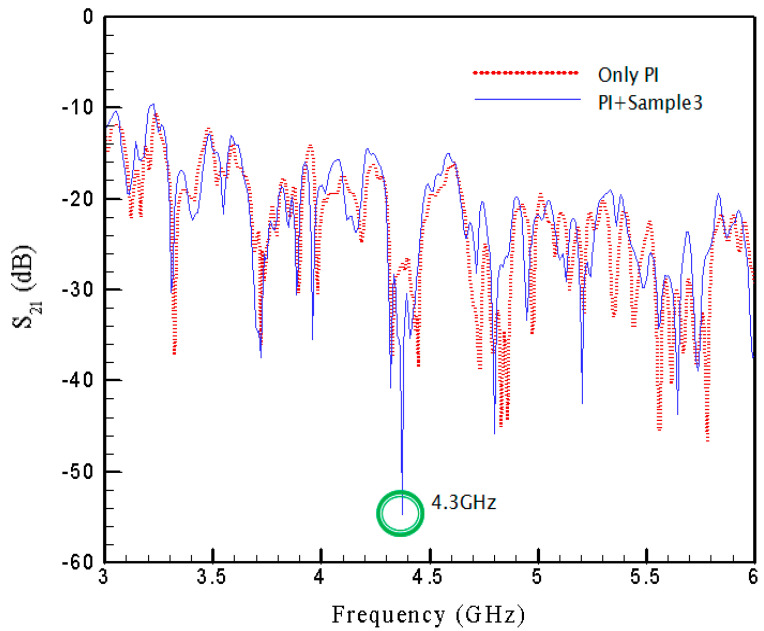
Transmission coefficient with SRR3.

Similar results for SRR3 with pattern size of 7 mm are conveyed in Figure 10. It is clearly seen that the negative peak of the transmission coefficient is −54.7 dB at 4.3 GHz and the maximum absorptivity is 99.8% at this frequency.

**Figure 11:**
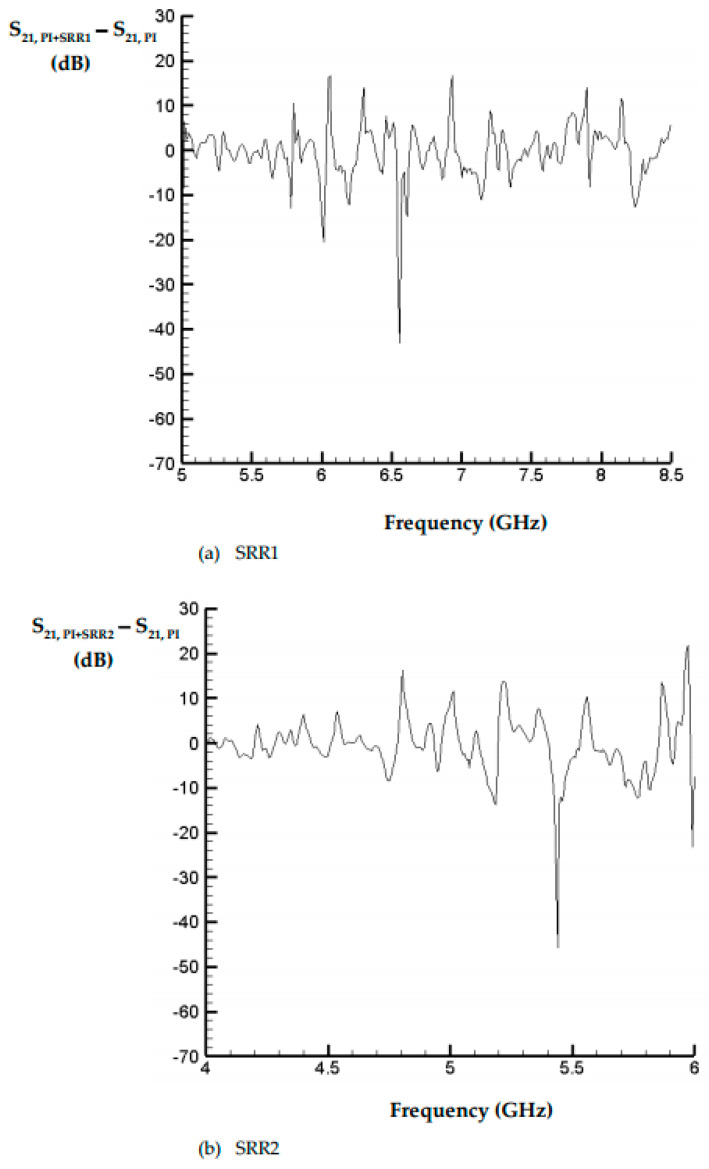

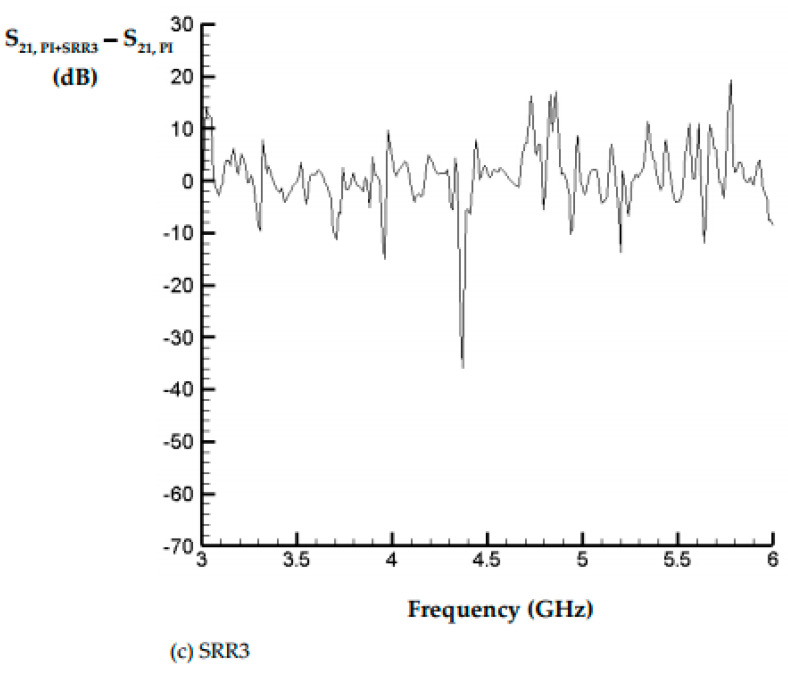
Difference between PI film with and without SRRs.

In Figure 8, Figure 9 and Figure 10, the spectrums corresponding to the pure PI film without SRRs are also provided using the red-dot curves in respective figures to show the influences of the existence of SRRs. One thus can see clearly the contribution from the SRR structure itself. The separate plots may be helpful for the difference between the PI film with and without SRRs. Therefore, In Figure 11, three plots, Figure 11a–c, are provided to display the difference for cases with SRR1, SRR2 and SRR3, respectively.

Table 3 summarizes the comparison in operation frequency between the numerical and the experimental data. Again, it is seen that an increase in pattern size results in a decrease in the operation frequency. In this table, the trends of the two sets of data closely agree with each other.

## 6. Conclusions

A cost-effective and efficient fabrication method for the flexible metamaterial structures with high electromagnetic wave absorptivity is developed and tested in this study. The structure mainly consists of a flexible dielectric layer made of polyimide (PI) and an array of split-ring resonators. The split-ring resonators of different geometric dimensions can be easily printed on the PI layer by using a silver nanoparticles ink jet printer. Therefore, the costly fabrication process for the split-ring resonators can be replaced with printing method. Numerical simulation and experiments on the absorption characteristics of the film are performed within the range from 2.0 to 9.0 GHz.

It is noticed that an adjustment in the geometric parameters could alter the operation frequency of the metamaterial structure. An increase in pattern size results in a decrease in the operation frequency. For investigating the size effects, three baseline split-ring resonators, SRR1, SRR2 and SRR3, are fabricated and measurements are conducted. Experimental results show that as the pattern size L_p_ is increased from 5 to 7 mm, the operation frequency with higher absorptivity is decreased from 6.6 to 4.3 GHz. For the three particular cases, the absorptivity can reach 96.6~99.8%. A comparison between the numerical and experimental data shows that the numerical predictions of the operation frequency with higher absorptivity closely agree with the experimental data.

However, when more dimensions, such as C, D or W, are taken into consideration, numerical predictions show that the operation frequency may increase with these dimensions. It implies that the combined influence of the geometric parameters is rather complicated and further study of this issue is still necessary.

## Figures and Tables

**Table 1:** Numerical predictions of electromagnetic fields for the test cases.

	L_p_= 5 mm at 6.5 GHz (SRR1)	L_p_= 6 mm at 5.3 GHz (SRR2)	L_p_= 7 mm 4.3 GHz (SRR3)
Magnitude of electric field intensity [V/m]	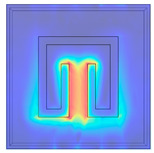	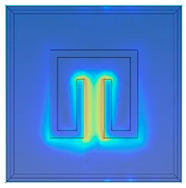	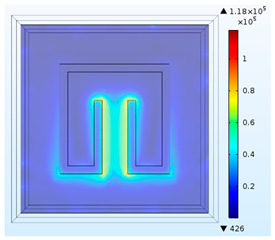
Magnitude of magnetic field intensity [A/m]	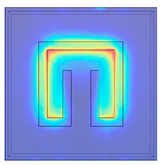	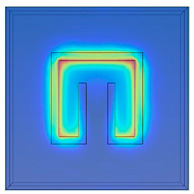	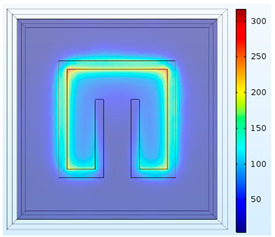
Magnitude of magnetic energy density [J/m^3^]	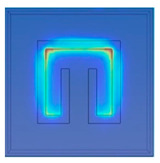	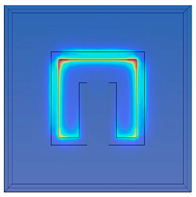	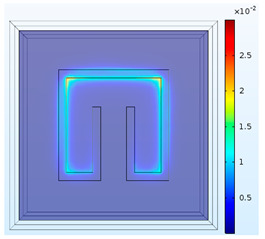

**Table 2:** Effects of geometric parameters on operation frequency.

L_p_ (mm)	C (mm)	D (mm)	W (mm)	Operation Frequency (GHz)
5	1	1.16	0.5	5.8
6	1	1.16	0.5	4.7
7	1	1.16	0.5	4.2
7	1	1.16	0.5	4.3
7	2	1.16	0.5	4.4
7	3	1.16	0.5	4.6
7	5	1.16	0.5	4.8
7	2	1.44	0.5	4.4
7	2	1.64	0.5	4.5
7	2	2.04	0.5	4.7
7	2	1.16	0.5	4.3
7	2	1.16	0.6	4.4
7	2	1.16	0.7	4.5

**Table 3:** Comparison between numerical and experimental data in operation frequency.

Case	Operation Frequency [GHz]	
	Numerical Simulation	Experiments
SRR1	6.5	6.6
SRR2	5.3	5.4
SRR3	4.3	4.3

## Data Availability

The study did not report any data.
